# Dissipation of mesoscale eddies and its contribution to mixing in the northern South China Sea

**DOI:** 10.1038/s41598-018-36610-x

**Published:** 2019-01-24

**Authors:** Qingxuan Yang, Maxim Nikurashin, Hideharu Sasaki, Hui Sun, Jiwei Tian

**Affiliations:** 1 Physical Oceanography Laboratory/CIMST, Ocean University of China, and Qingdao National Laboratory for Marine Science and Technology, Qingdao, China; 2 ARC Centre of Excellence for Climate Extremes, Sydney, Australia; University of Tasmania, Hobart, Australia; 3 Application Laboratory, JAMSTEC, 3173-25 Showa-machi, Kanazawa-ku, Yokohama, Kanagawa 236-0001 Japan

## Abstract

It is reported that turbulent mixing is enhanced in the South China Sea (SCS), and it is highly variable in both space and time. Generation and breaking of internal tides has been identified as the main process to drive turbulent mixing in the SCS, while the contributions from other processes are not clear enough. Here we investigate the potential contribution from mesoscale eddies to turbulent mixing in the SCS using a high resolution numerical simulation. Our results show that mesoscale eddies in the SCS effectively dissipate over complex rough topography and indicate that the generation of submesoscale motions and lee waves are two pathways for the transfer of mesoscale eddy energy down to small dissipation scales. The energy loss from mesoscale eddies near the Xisha Islands is estimated to be sufficient to sustain turbulent kinetic energy dissipation rate of *O* (10^−8^) W/kg. This study suggests an alternative and potentially efficient mechanism to internal tides for the local maintenance of turbulent mixing in the SCS.

## Introduction

The South China Sea (SCS) is a large marginal sea, which is connected with the Pacific Ocean through the Luzon Strait. There are prominent topographic features in the SCS, such as the Xisha and Nansha Islands, which are made up of several islets and have typical small-scale topography. In the SCS, there are also abundant dynamical processes with different spatial scales, including mean currents^1^, mesoscale eddies^2^, internal waves^3^, and turbulent mixing^4^. Turbulent mixing in the SCS has been suggested to have a strong impact on the local water mass transformation and hence on the global overturning circulation in the Pacific Ocean^5^. Turbulent mixing occurs at a centimeter scale, and must be fed with a supply of energy from upscale dynamics. However, processes and energy pathways maintaining mixing in the SCS are not clear enough. 

Our understanding of turbulent mixing in the SCS has been improved in the past years. Studies of spatial structure and temporal variability of turbulent mixing in the SCS have been carried out successively^4,6–9^. These studies revealed that enhanced mixing with diapycnal diffusivity of 10^−3^ m^2^/s, being two orders of magnitude larger than that in the Pacific Ocean, occurs in the deep SCS and it is highly variable in both space and time. Several processes causing this high-level mixing have been proposed, such as internal solitary waves responsible for the elevated mixing over the continental shelf^10–13^ and tidally generated lee waves supporting enhanced mixing in the near bottom water^14^. Among these processes, contribution from internal tides to the turbulent mixing in the SCS was well established. Internal tides, generated from the Luzon Strait and propagating westward into the SCS, play a key role in furnishing elevated mixing in the northern SCS. Based on field experiments^3,6,15^ and numerical simulations^16,17^, the energy flux of internal tides entering the SCS was estimated to be in the range of 7–10 GW. However, a fraction (such as reaching 20%^18^) of this energy flux is used to provide energy for generating internal solitary waves, which propagate to the northern continental shelf and eventually dissipate over shallow waters^19^.

Moreover, Zhao^19^ examined the internal tide behavior in the SCS and found that internal tides generated in the Luzon Strait weakened dramatically in the central SCS and were hardly detected in the southern SCS. Therefore, other alternative processes for maintaining turbulent mixing in the SCS should be explored. Mesoscale eddies are ubiquitous in the SCS and particularly the southern SCS is a hotspot of eddy occurrence and genesis^20^. It is reasonable to expect that the dissipation of mesoscale eddy energy is a possible mechanism to maintain turbulent mixing in the SCS. Although a few studies tried to explore the influence of mesoscale eddies on the turbulent mixing in the SCS^21,22^, the relationship between eddy dissipation and mixing are needed to be improved. The goal of this study is to inspect possible contribution of mesoscale eddies to small scale mixing using numerical simulation with a high horizontal resolution of 1/30° (see Methods).

## Results

### Eddy propagation

**Figure 1:**
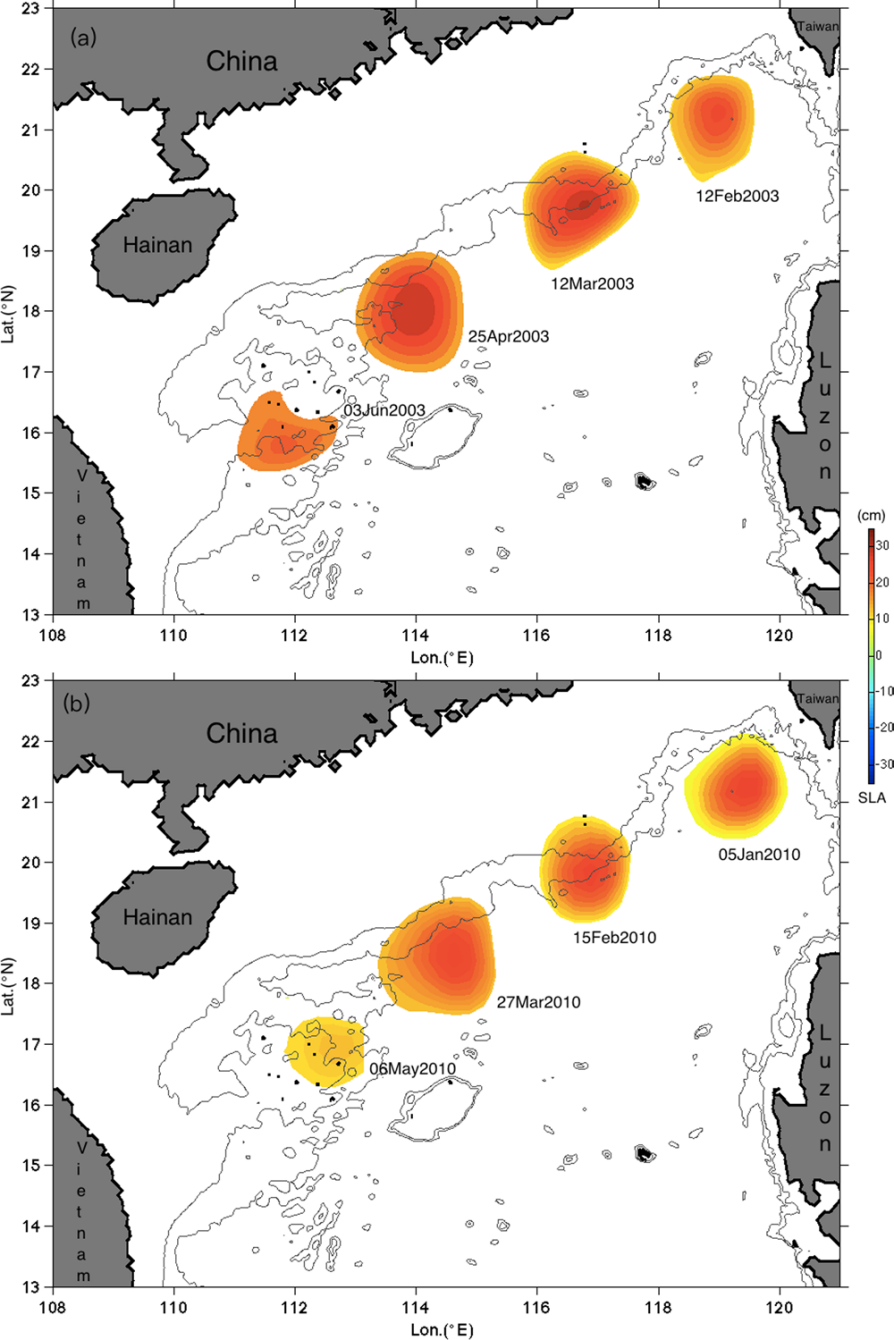
Propagation and dissipation of eddies in the SCS. Sea level anomaly from OFES simulation (**a**) and altimeter observation (**b**). The dates are labeled individually for each eddy. The color shading indicates SLA. The contours are isobaths of 1000 and 2000 m. The figure was made using MATLAB R2016b (http://www.mathworks.com).

We first examine mesoscale eddy propagation using the sea level anomaly (SLA) (see Methods) from 2001 to 2003. As seen from the temporal variation of SLA, an anticyclonic eddy starting southwest of Taiwan Island on February 12, 2003 propagated southwestward until it mostly dissipated near the Xisha Islands on June 03, 2003 (Fig. 1a). The propagation path followed closely the continental slope, i.e., it was basically following the isobath of 1000 m. The propagation distance reached as far as 1000 km, and the duration of the propagation was about three months and three weeks, which correspond to the eddy propagation speed of about 10.5 cm/s. This propagation speed is comparable to the phase speed of 7.0 cm/s estimated for a free long Rossby wave of the first baroclinic mode^23^. By examining the eddy size enclosed by the outermost contour of SLA, the eddy experienced no significant shape transformation along the propagation path before meeting the Xisha Islands.

To validate this model result, we examined the altimeter observation (CMEMS product) from 1993 to 2016 and checked if there was similar eddy evolution. We are able to identify the same eddy process seven times with propagation distance exceeding 500 km (Table S1). As an example, an anticyclonic eddy existed southwest of Taiwan Island on January 5, 2010 is illustrated in Fig. 1b. In the following four months, it moved southwest along the continental slope, and started to dissipate when it approached the Xisha Islands on May 6, 2010. The distance travelled by this eddy was about 970 km and the speed was about 9.3 cm/s, which compared well with the eddy evolution in the model (Fig. 1a). This comparison validates the model performance and the total flow decomposition (see Methods). More information about other observed eddies on different dates is given in Table S1.

**Figure 2:**
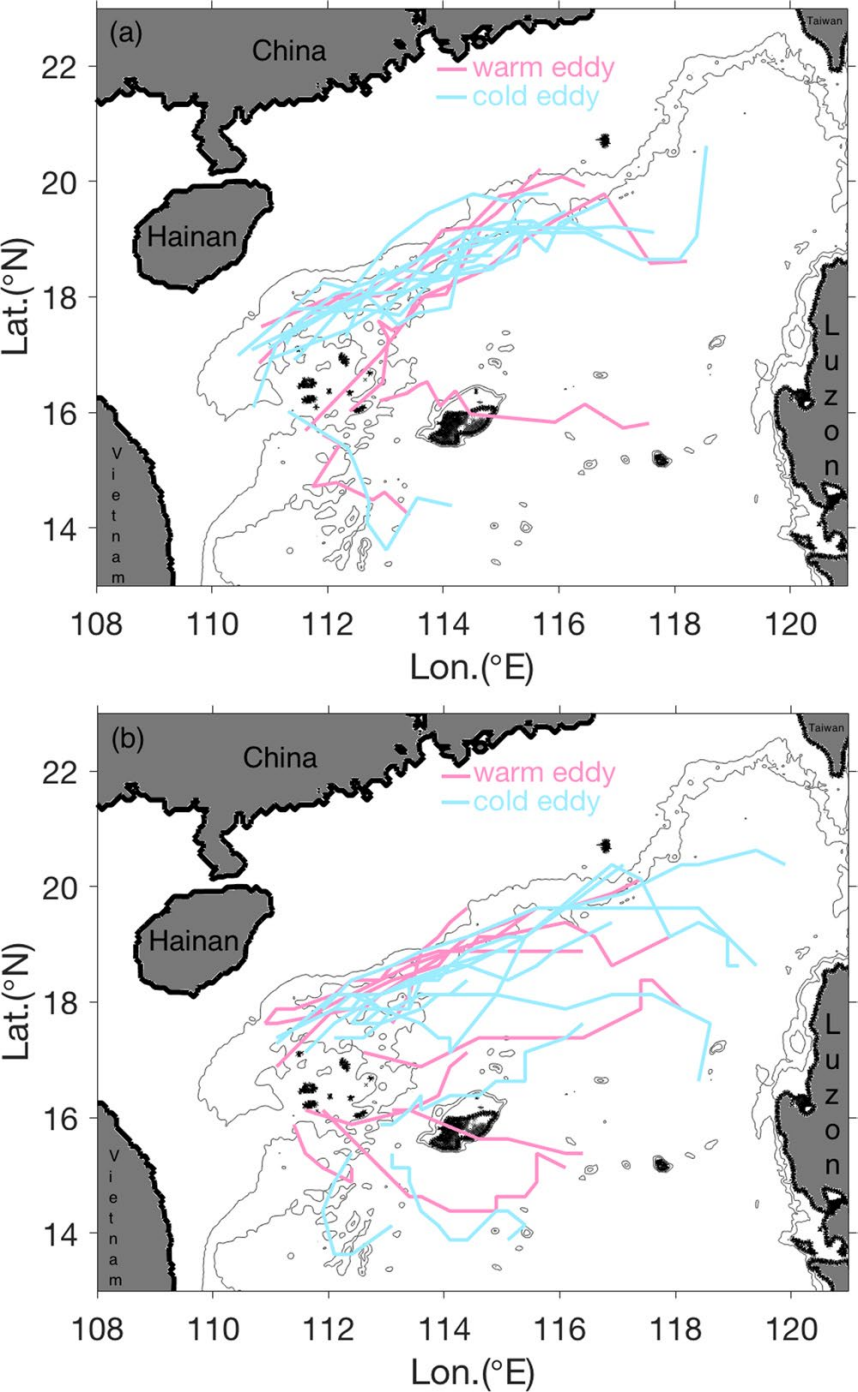
Tracks of eddies dissipating around the Xisha Islands from 2001 to 2003. Panel (a) is derived from the model output, and panel (b) is from the altimeter observation. The figure was made using MATLAB R2016b (http://www.mathworks.com).

Apart from the eddy coming from the southwest of Taiwan, we also examined the eddies starting from other locations and dissipating around the Xisha Islands based on the model output and altimeter observation from 2001 to 2003 (Fig. 2). The results show that there are in total 19 eddies detected from the model output and 22 eddies from the altimeter observation losing most of their kinetic energy around the region of Xisha Islands. Among these eddies, 7 (10) are warm- and 12 (12) are cold-core eddies for the simulated (observed) results. Most eddies came from the northeastern SCS with their propagation track following the continental slope (Fig. 2), consistent with the eddy trajectory shown in Fig. 1. The duration of these eddies ranges from 5 to 22 weeks. On average, the Xisha Islands experienced eddy energy dissipation once every two months. It is notable that this frequency of eddy dissipation may be underestimated, because some eddies generated in the deep layer have no surface signals^24^, which cannot be identified adequately from the altimeter data. Also, some eddies with smaller scales cannot be well resolved in the model output. This implies that the Xisha Islands is a highly effective location to dissipate eddy energy.

### Eddy dissipation

**Figure 3:**
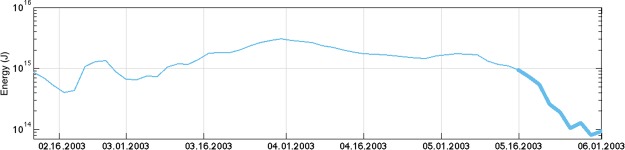
EKE variation along the eddy propagation path. The thick curve indicates the energy dissipation when the eddy reached the Xisha Islands. A corresponding depth-time variation of EKE along the eddy moving path is given in Fig. S1.

Although altimetry reveals the trajectory and evolution of SLA of eddies over a long period, it cannot be used to estimate the energy of mesoscale eddies because the full-depth velocity information is not available. We now examine the evolution of the kinetic energy (EKE; see Methods) along the eddy travelling path for the eddy shown in Fig. 1a using the outputs of the high-resolution numerical model. The result, shown in Fig. 3, suggests that the eddy intensity varied slightly before approaching the Xisha Islands. The EKE level was mostly on the order of 10^15^ J before May 16, and the maximum value reached 3 × 10^15^ J on April 1. After May 16, the EKE decreased sharply, from 10^15^ J to less than 10^14^ J on June 1. On May 16, the southwestern periphery of the eddy just met the Xisha Islands. Due to the southwestward movement, on June 1, the eddy completely covered the Xisha Islands. During this process, as much as 1.6 × 10^15^ J kinetic energy was lost around the Xisha Islands, which is consistent with the result from the SLA field, suggesting that the Xisha Islands is a highly effective location for EKE loss.

To better understand the eddy energy loss in the region of Xisha Islands, we estimated the EKE budget over the period from May 16 to June 1 and over a fixed volume around the Xisha Islands (see Methods). The rate of the total EKE loss reaches as high as 1.11 GW. Out of this energy loss, the outward transport across the lateral boundaries was 0.13 GW, the dissipation due to bottom drag at the bottom boundary was 0.25 GW, and the negative wind stress work at the surface boundary was 0.11 GW. The remaining value of the energy loss, reaching about 0.62 GW or 56% of the total EKE loss, is dissipated within the volume of the eddy and hence will eventually transfer to small dissipation scale and power turbulent mixing. We have applied this EKE budget analysis to each eddy from 2001 to 2003 shown in Fig. 2a, and the result suggests that, on average over this period, 0.27 GW of the EKE was lost to dissipation scale in the Xisha Islands.

Assuming that this energy is dissipated uniformly over the volume of the Xisha Islands region of 2.4 × 10^13^ m^3^ and using the squared buoyancy frequency of 1.8 × 10^−5^ 1/s^2^ in this region, we can estimate that the energy dissipation will result in a turbulent kinetic energy dissipation rate of 1.1 × 10^−8^ W/kg and a corresponding eddy diffusivity of 1.2 × 10^−4^ m^2^/s (see Methods). However, if this energy is not uniformly redistributed in the vertical and more of the dissipation take place in the deep layer, such as 500 m above the bottom, then stronger dissipation rate and diffusivity values of 2.0 × 10^−8^ W/kg and 8.9 × 10^−4^ m^2^/s are expected using the squared buoyancy frequency of 4.5 × 10^−6^ 1/s^2^. This estimate is comparable to the turbulence level which is caused by internal tides in the northern SCS, and to the observed levels of turbulent energy dissipation in the region of the Xisha Islands^4^. This implies that mesoscale eddies in the Xisha Islands region can contribute sufficient amount of energy to the small dissipation scales to power turbulent mixing.

Next, we explore the processes via which the mesoscale eddies can potentially transfer its energy to small dissipation scales. Two possible candidates, which have been considered as effective energy transferring routes between mesoscale eddies and dissipation scales, are the generation of submesoscale currents in the upper ocean and lee waves in the deep ocean.

### Generation of submesoscale currents

McWilliams^25^ showed that submesoscale motions provide a dynamic conduit for energy transfer toward microscale dissipation and diapycnal mixing, and pointed out that one of the main submesoscale generation mechanisms near islands is topographic wake. Based on advanced synthetic aperture radar images, Zheng *et al*.^26^ found submesoscale eddy trains occurred northwest of Babuyan Islands, being located in the southern tip of Luzon Strait, and suggested that the small island favors the generation of submesoscale motions. Recently, it is reported that the periphery of mesoscale eddies is favorable for the occurrence of submesoscale currents^22,27^. The Xisha Islands are made up of several such islets, where the mesoscale eddies are rich in the same time.

**Figure 4:**
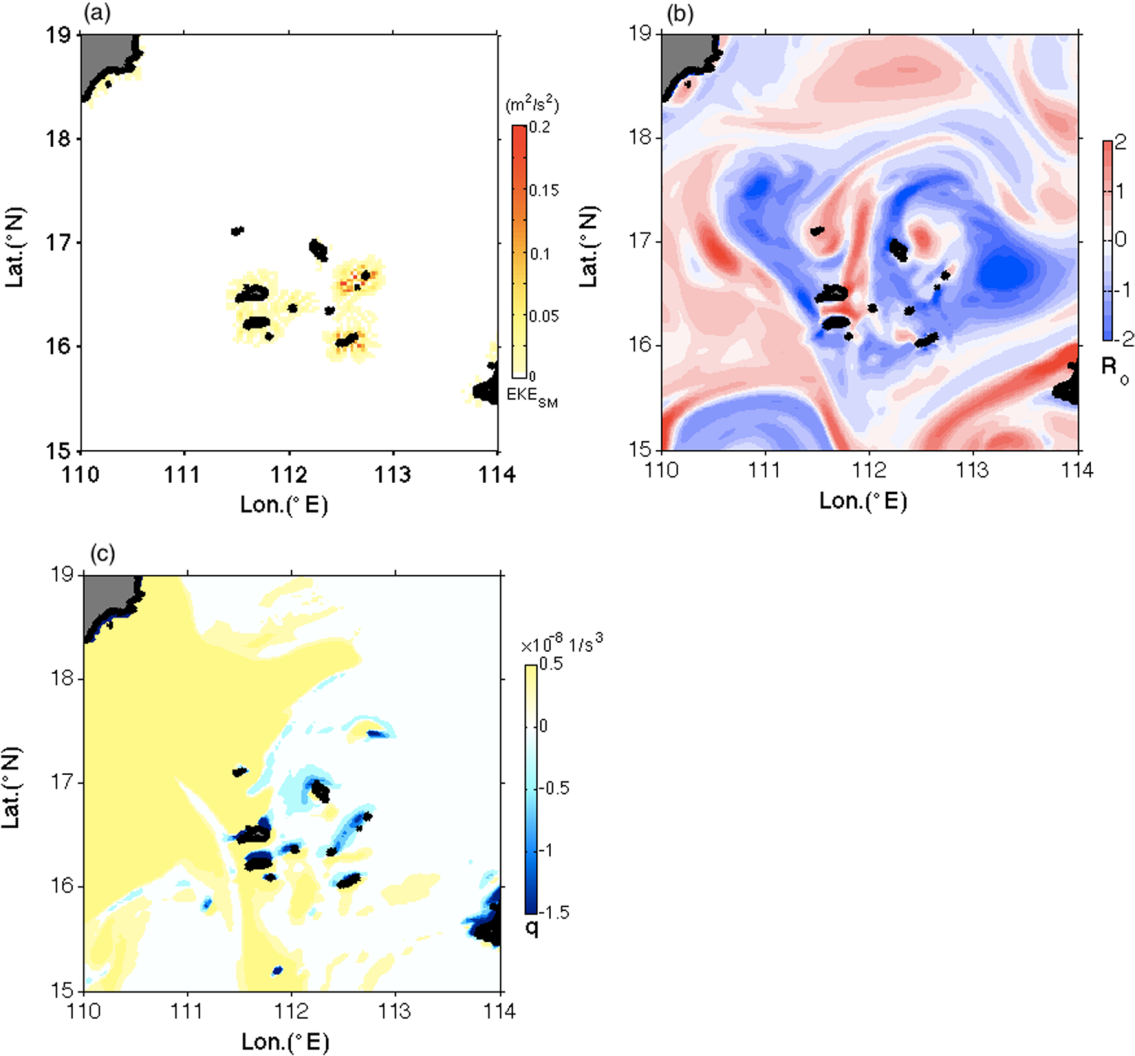
Submesoscale currents performance around the Xisha Islands. Panels (a–c) show maps of kinetic energy of submesoscale currents, Rossby number, and Ertel potential vorticity at 3 m on May 28, 2003. The figure was made using MATLAB R2016b (http://www.mathworks.com).

The submesoscale velocity field estimated from the model shows that very active submesoscale currents occurred around the Xisha Islands, with associated kinetic energy reaching 0.2 m^2^/s^2^ (Fig. 4a), which came at the expense of mesoscale eddy decay. The Rossby number (*R*_*o*_; see Methods) map around the Xisha Islands gives a supporting evidence that there exist abundant submesoscale currents (Fig. 4b). The results show a clear pattern of significantly elevated *R*_*o*_ values reaching unity around all islets and appearing as filaments, indicating strong ageostrophic behavior of submesoscale currents. This agrees well with the topographic wake mechanism.

An additional evidence of the submesoscale motions is shown by the map of Ertel potential vorticity (EPV; see Methods) (Fig. 4c). The vigorous submesoscale activity can be illustrated by the EPV field when eddy reached the Xisha Islands, where EPV had strong negative values reaching −1 × 10^−8^ 1/s^3^ around the islets. These negative values shown as small patches and filaments were comparable to those in the west boundaries of North Atlantic Ocean and North Pacific Ocean, where intensified submesoscale currents exist^28,29^. The negative EPV values indicated that the mesoscale currents could be subject to the submesoscale instabilities, such as centrifugal instability or symmetric instability^30^, which play as mediators in the energy dissipation of mesoscale currents as they can drive a forward energy cascade from mesoscale to smaller scales^31,32^.

### Generation of Lee Waves

Besides submesoscale currents, there is another effective way to transfer the mesoscale energy toward smaller scales near complex topography, which is the interaction between mesoscale eddies and small-scale bathymetry, and is more complicated than being driven by barotropic tides^14^. Through this interaction, lee waves can be effectively generated, if small-scale bathymetry in the lee wave radiating wavelength range is rough^33,34^. Lee wave generation is not resolved in our model and therefore, in this study, we examine the lee wave generation conditions to check whether the generation of lee waves is a possible candidate to drive turbulent mixing near the Xisha Islands.

**Figure 5:**
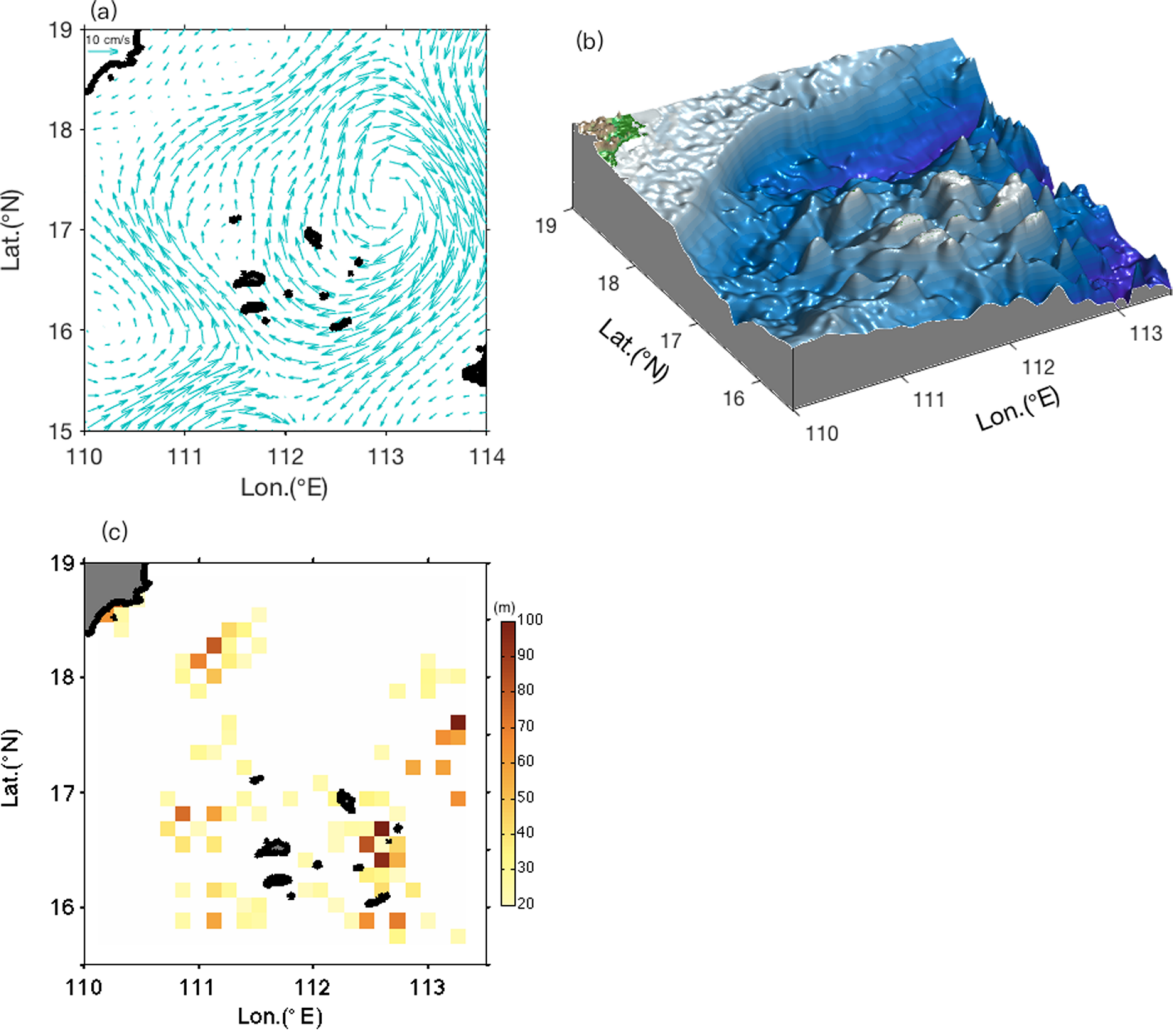
Mesoscale currents and topography feature around the Xisha Islands. Panel (a) shows mean velocity field of mesoscale currents within 300 m above the bottom of Xisha Islands, (**b**) bathymetry of the Xisha Islands, and (**c**) topographic roughness calculated within the lee waves radiating wavelength range. The figure was made using MATLAB R2016b (http://www.mathworks.com).

The magnitude of mesoscale flow was strong with a mean value of 5 cm/s near the bottom of Xisha Islands (Fig. 5a). According to the linear theory, a geostrophic flow with bottom velocity of *U*_*b*_ and bottom stratification *N*_*b*_ can radiate lee waves from topography with horizontal wavenumber *k*_*T*_ in the range $\frac{f}{{U}_{b}} < {k}_{T} < \frac{{N}_{b}}{{U}_{b}}$. Using *U*_*b*_ of 5 cm/s and *N*_*b*_ of 2 × 10^−3^ 1/s, values averaged within 300 m above bottom, we obtained the radiating wavelength range. Figure 5c shows the topographic roughness estimated in the lee wave radiating wavelength range using the local bathymetry (Fig. 5b) (see Methods). The result suggests that the Xisha Islands have favorable bottom bathymetry for the lee wave generation, with maximum roughness reaching 100 m around several islets of the Xisha Islands. For comparison, in the Drake Passage region of the Southern Ocean, which is reported as a hotspot of lee waves generation^35,36^, the mean value of roughness is about 60 m, the same level as that of the Xisha Islands.

## Discussion

Our results show that the amount of EKE transferred to dissipation scales around the Xisha Islands reaches a mean value of 0.27 GW. In the southern SCS, there are lots of islets like the Xisha Islands; they are named the Nansha Islands, which are potentially hot spots for dissipating EKE and hence furnishing turbulent mixing. Internal tide energy flux generated in the Luzon Strait is not totally used to furnish turbulent mixing in the SCS deep water region; a fraction of this energy flux is used to provide energy for generating internal solitary waves, which propagate to the northern continental shelf and eventually dissipate over shallow waters. For example, more than half of the semidiurnal internal tide energy flux is used for internal solitary wave generation^17,19^. Moreover, the dissipation of mesoscale eddy energy is especially important for supporting turbulent mixing in the southern SCS, because the internal tides generated in the Luzon Strait are hardly detected in the southern SCS^19^, which, in contrast, is a hotspot of eddy occurrence and genesis^20^. Therefore, mesoscale eddy is potentially an alternative candidate to internal tide for the local maintenance of mixing in the SCS.

Our results also suggest that the generation of submesoscale currents and that of internal lee waves are two effective pathways of transferring mesoscale eddy energy to dissipation scales. In this study, the generation of submesoscale currents over the Xisha Islands seems to be a combined effect of small-scale topography wake and mesoscale eddies, which resulted in an enhanced submesoscale motions and different to those found in other regions^26,31^. Lee waves over the Xisha Islands are mainly from the interaction between the active geostrophic flow and complex bathymetry, which are unlike those driven by barotropic tides^14^. The Xisha Islands is a region where submesoscale motions and lee waves can occur at the same time. Submesoscale currents and lee waves can bridge the scale gap between mesoscale eddies and turbulent mixing, and make the eddy energy forward cascade possible. These contributions of mesoscale eddies to turbulent mixing are missing in the general circulation model, and need to be parameterized. In this study, lee waves are not resolved due to the model spatial resolution. The energetics of submesoscale currents and internal lee waves are difficult to estimate accurately, which would require models with a higher spatial resolution and field experiments those are comprehensively designed.

## Methods

### Model

In this study, the simulation output from the Ocean general circulation model For the Earth Simulator (OFES) was used. The OFES was extended by Sasaki and Klein^37^ from a mesoscale eddy-resolving of 1/10° to 1/30°, which could partially capture the signals of submesoscale currents. The model has 100 layers in the vertical, and the domain ranges from 100°E to 70°W zonally and from 20°S to 66°N meridionally. The initial field is 1/10° simulation output of January 1, 2000 and the forcing fields includes 6-hourly surface wind stress and heat flux of the Japanese 25-yr Reanalysis data^38^ (JRA-25). For horizontal mixing of momentum, a biharmonic operator is applied to reduce numerical noise. Biharmonic viscosity coefficient is 1.0 × 10^9^ m^4^/s. For vertical mixing, the scheme of Noh and Kim^39^ is used. The simulation is integrated from year 2000 to 2003, and the output covers the northern SCS region (108–124°E, 13–24°N). The model is run without data assimilation to observation, and the daily outputs are horizontal velocities (*u*, *v*), sea surface height (*η*), temperate (*T*) and salinity (*S*).

### Flow decomposition

First, we estimate the mean flow as a temporal average over 90 days, namely, $\bar{{\boldsymbol{u}}}(t)=\frac{1}{90}{\int }_{t-45}^{t+45}{\boldsymbol{u}}(t)dt$. Then, the mean flow is removed from the total flow, and a lowpass filter and a highpass filter with the cutoff wavelengths of 100 km and 10 km, respectively, are applied to this residual flow. The lowpass and highpass filtered velocities are considered as mesoscale velocities (*u*′, *v*′) and submesoscale velocities (*u*_*sm*_, *v*_*sm*_), respectively. Similarly, this operation is conducted for density, which is estimated based on temperature and salinity. As an example, the results of the flow decomposition are shown in Fig. S3a, where the mesoscale field appears to be well resolved with dominant eddies, and submesoscale currents are especially developed around the periphery of the mesoscale eddies although their magnitudes are much weaker than those of mesoscale structures. Figure S3b indicates that the submesoscale currents are weak when there is no eddy around the Xisha Islands.

### Sea level anomaly calculation

Sea level anomaly (SLA, *ζ*) is the deviation from the mean state of sea surface height (SSH, *η*), as in the formula $\zeta =\eta -\frac{1}{90}{\int }_{t-45}^{t+45}\eta (t)dt$. To determine how long the time should be used to make the average, the *ζ* and *η* data from altimeter observations are analyzed in advance as a guidance. For an *ζ* field at one day, such as on January 05, 2010 in Fig. S4a, we compared it with the computed *ζ* field obtained by subtracting the mean value from the altimeter observed *η* field on the same day. Different periods of averaging time were tested, spanning from 1 month to 5 months; and we found the most proper time period is 3 months (Fig. S4d), with a minimum root mean square value of 6.3 cm. The computed *ζ* field shares the same pattern and magnitude as the observed. Based on this, the *ζ* field was constructed with the *η* field of model output, which shows dominant signals of mesoscale motion, indicating this estimation is reasonable. This derived *ζ* field is then used to detect and track the eddy.

### Eddy detection

There are several existing eddy detection methods, physical parameter based, flow geometry based, and the combination of the two methods^40,41^. Using the Okubo-Weiss (OW) parameter, the Okubo-Weiss algorithm^42,43^ is one of the most widely used detection algorithms. However, the extra noise in the OW field induced by velocity derivatives sometimes makes it difficult to identify the eddy boundary. In this study, for simplicity we use SLA to identify the eddies and use stream function computed from mesoscale velocity field to define the eddy boundaries at different depths. As a comparison, the eddy boundary is also determined through the outermost enclosed contours of both SLA and stream function in the surface layer (Fig. S5), which suggests a good agreement between them.

### Eddy kinetic energy and its budget

Eddy kinetic energy (EKE) is a volume integrated value, which is integrated with depth (*H*) and area (*Ω*) occupied by the eddy,

1 $${\rm{EKE}}={\rho }_{0}{\int }_{-H}^{0}\int {\int }_{{\rm{\Omega }}}\frac{1}{2}(\overline{{u^{\prime} }^{2}}+\overline{{v^{\prime} }^{2}})dsdz$$

where *ρ*_0_ is a reference value of seawater density.

The EKE budget is expressed as

2 $$\begin{array}{c}\frac{\partial }{\partial t}{\rm{EKE}}=Transport+Mean\,to\,EKE+EPE\,to\,EKE+Wind\,Stress+Bottom\,Drag+Dissipation\end{array}$$

where *Transport* is

$$-{\rho }_{0}{\int }_{-H}^{0}\int {\int }_{{\rm{\Omega }}}\frac{\partial (\frac{1}{2}\overline{{u}_{j}}\overline{{u^{\prime} }_{i}^{2}}+\frac{1}{2}\overline{{u^{\prime} }_{j}{u^{\prime} }_{i}^{2}}+\frac{1}{{\rho }_{0}}\overline{{u^{\prime} }_{j}p^{\prime} })}{\partial {x}_{j}}dsdz,$$

*Mean to EKE* conversion is

$$-{\rho }_{0}{\int }_{-H}^{0}\int {\int }_{{\rm{\Omega }}}\overline{{u^{\prime} }_{j}{u^{\prime} }_{i}}\frac{\partial \overline{{u}_{i}}}{\partial {x}_{j}}dsdz,$$

*EPE to EKE* conversion is

$${\rho }_{0}{\int }_{-H}^{0}\int {\int }_{{\rm{\Omega }}}\overline{w^{\prime} b^{\prime} }dsdz,$$

*Wind Stress* work is

$$\int {\int }_{{\rm{\Omega }}}\overline{\mathop{{{\boldsymbol{\tau }}}_{{\boldsymbol{w}}}}\limits^{\rightharpoonup }\cdot \mathop{{{\boldsymbol{v}}^{\prime} }_{{\boldsymbol{top}}}}\limits^{\rightharpoonup }}ds,$$

*Bottom Drag* work is

$$-\int {\int }_{{\rm{\Omega }}}\mathop{{{\boldsymbol{\tau }}}_{{\boldsymbol{d}}}}\limits^{\rightharpoonup }\cdot \mathop{{{\boldsymbol{v}}^{\prime} }_{{\boldsymbol{bot}}}}\limits^{\rightharpoonup }ds,$$

*Dissipation* is

3 $$EK{E}_{dis}={\rho }_{0}{\int }_{-H}^{0}\int {\int }_{{\rm{\Omega }}}-{A}_{v}\bar{{(\frac{{\rm{\partial }}{u{\rm{^{\prime} }}}_{i}}{{\rm{\partial }}z})}^{2}}-{A}_{h}\bar{{(\frac{{\rm{\partial }}{u{\rm{^{\prime} }}}_{i}}{{\rm{\partial }}{x}_{j}})}^{2}}dsdz,$$

where *i* = (1, 2) and *j* = (1, 2) in the transport term since the contribution from *u*_3_ (*w*) is small and neglected, *p* is pressure, $b=-g\frac{\rho }{{\rho }_{0}}$ is buoyancy, *A*_*v*_ (*A*_*h*_) is vertical (horizontal) eddy viscosity, and the prime denotes mesoscale eddy induced component. Moreover, we did not take the terms *Mean to EKE* and *EPE to EKE* into account, because the contributions of these two terms are relatively small in the present case that the eddy is far from the generation site, and hence are often omitted. Figure S6 indicates that mean kinetic energy has almost no change when the eddy dissipates its energy near the Xisha Islands, with a mean value of ~5 × 10^13^ J. For example, based on the observation, Zhang *et al*.^44^ found *Mean to EKE* conversion was approximately 1/80 of the dissipation term. As for the *EPE to EKE* conversion, Gula *et al*.^28^ suggested it is much smaller than the dissipation term by examining some numerical simulation output. More importantly, Nikurashin *et al*.^45^ show that it is mainly dissipation in the interior that sinks energy of mesoscale eddies. $\mathop{{{\boldsymbol{\tau }}}_{{\boldsymbol{w}}}}\limits^{\rightharpoonup }$ $\mathop{({{\boldsymbol{\tau }}}_{{\boldsymbol{d}}})}\limits^{\rightharpoonup }$ is surface wind (bottom drag) stress, and $\mathop{{{\boldsymbol{v}}^{\prime} }_{{\boldsymbol{top}}}}\limits^{\rightharpoonup }$ ($\mathop{{{\boldsymbol{v}}^{\prime} }_{{\boldsymbol{bot}}}}\limits^{\rightharpoonup }$) is the eddy velocity at the sea surface (bottom). Here, $\mathop{{{\boldsymbol{\tau }}}_{{\boldsymbol{w}}}}\limits^{\rightharpoonup }$ is from the JRA-25 momentum flux product,$\,\mathop{{{\boldsymbol{\tau }}}_{{\boldsymbol{d}}}}\limits^{\rightharpoonup }={\rho }_{0}{C}_{db}|\mathop{{{\boldsymbol{v}}^{\prime} }_{{\boldsymbol{bot}}}}\limits^{\rightharpoonup }|\mathop{{{\boldsymbol{v}}^{\prime} }_{{\boldsymbol{bot}}}}\limits^{\rightharpoonup },$ and *C*_*db*_ is the bottom drag coefficient with a value of 2.5 × 10^−3^^46^. When doing eddy energy budget through Equation (2), the terms we calculated using the model output were $\frac{\partial }{\partial {\rm{t}}}{\rm{EKE}}$, *Transport*, *Wind Stress* work, and *Bottom Drag* work, the terms we ignored were *Mean to EKE* and *EPE to EKE*, and the *Dissipation* term was then derived assuming that the eddy energy budget was in a balance state. The temporal variations of these terms over the period from May 16 to June 1 around the Xisha Islands are shown in Fig. S7.

### Estimation of eddy diffusivity

The dissipation rate *ε* is estimated as $\varepsilon =\frac{EK{E}_{dis}}{\rho V}$, where *V* is the water volume over which the energy $EK{E}_{dis}$ is dissipated, and *ρ* is density of sea water. The diffusivity *K* is derived from the formula $K=\Gamma \frac{\varepsilon }{{N}^{2}}$^47^. The vertical profile of squared buoyancy frequency *N*^2^ (calculated from model result) used here is shown in Fig. S8.

### Rossby number and Ertel potential vorticity

Rossby number (*R*_*o*_) is defined as the ratio of relative vorticity ($\partial v/\partial x-\partial u/\partial y$) to ambient vorticity (*f*). Ertel potential vorticity (EPV) is defined as $q=(f\,\hat{k}+\nabla \times \mathop{u}\limits^{\rightharpoonup })\cdot \nabla b$, where $\hat{k}$ is a unit vector in the vertical, and *b* is buoyancy.

### Bathymetry roughness

Bottom bathymetry roughness is estimated as integrating the two-dimensional bathymetry spectra within the lee waves radiating wavelength range. The topography data used to derive roughness is SRTM30_PLUS data with a resolution of 1/120°.

## Electronic supplementary material

Supplementary Information

## Data Availability

The altimeter products were downloaded from the CMEMS website at http://marine.copernicus.eu. The SRTM30_PLUS data were downloaded from the Satellite Geodesy website at http://topex.ucsd.edu/WWW_html/srtm30_plus.html. The OFES simulation output and computing codes used in this study are available on request to the authors. Matlab (R2016b) was used in generating all figures.
