# Supplementary material for: Dissipation of mesoscale eddies and its contribution to mixing in the northern South China Sea
Source: Sci Rep. 2019 Jan 24;9:556. doi: 10.1038/s41598-018-36610-x (PMC6345983; doi:10.1038/s41598-018-36610-x)
Supplement: Supplementary file 1 — Supplementary Information [file 41598_2018_36610_MOESM1_ESM.docx]

**Supplementary Information for**

**Dissipation of mesoscale eddies and its contribution to turbulent mixing in the northern South China Sea**

**Qingxuan Yang*^1^, Maxim Nikurashin^2^, Hideharu Sasaki^3^, Hui Sun^1^ & Jiwei Tian^1^**

^1^Physical Oceanography Laboratory/CIMST, Ocean University of China, and Qingdao National Laboratory for Marine Science and Technology, Qingdao, China

^2^ARC Centre of Excellence for Climate Extremes, Sydney, Australia; University of Tasmania, Hobart, Australia

^3^ Application Laboratory, JAMSTEC, 3173-25 Showa-machi, Kanazawa-ku, Yokohama, Kanagawa 236-0001, Japan


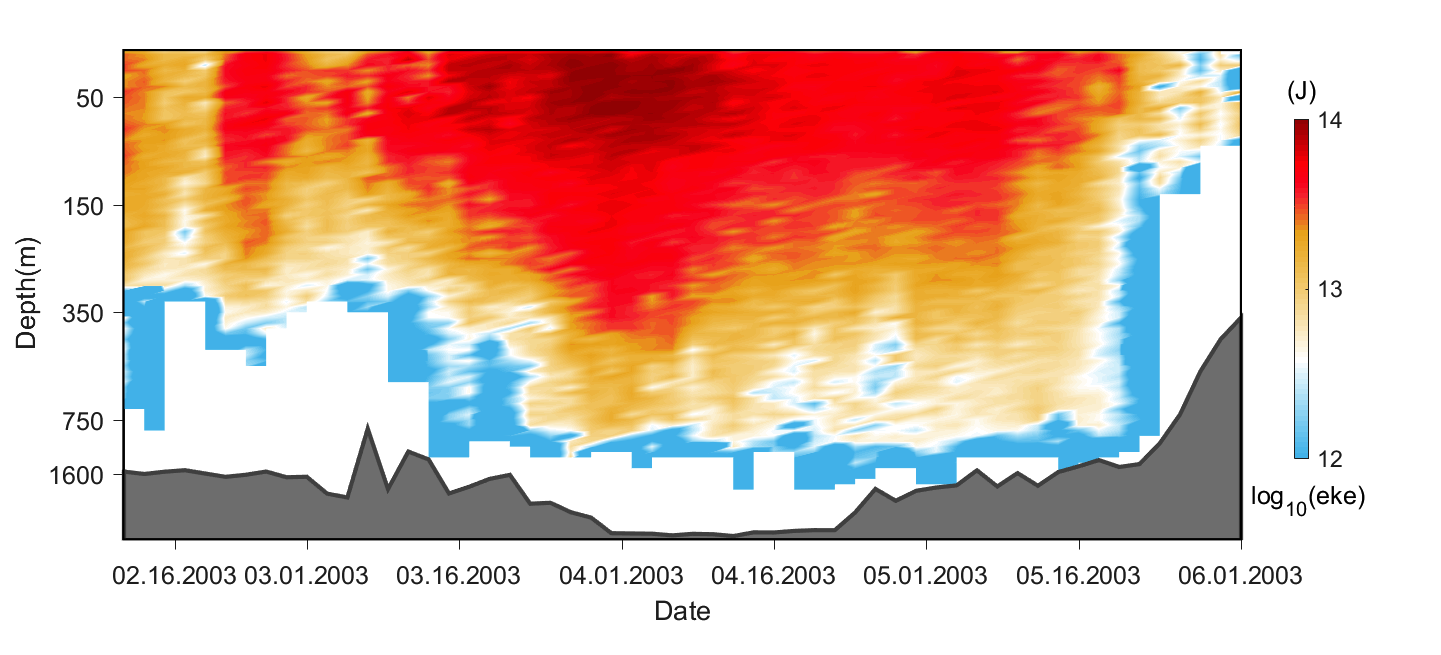


**Figure S1. Depth-time variation of EKE along the eddy moving path.** Here the logarithmic scale is used, and the gray shading indicates the bathymetry. The results show that EKE starts being dissipated dramatically in the whole water column when meeting the Xisha Islands on May 16. After covering the islands, the EKE level decreased sharply by 2 orders.


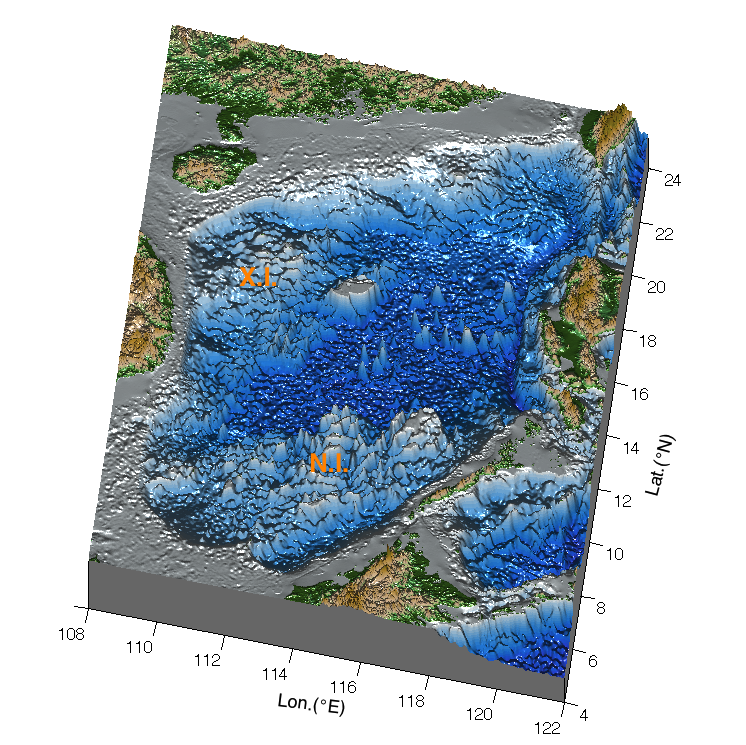


**Figure S2. Bathymetry of the South China Sea.** The Xisha Islands are located around 112°E, 16.5°N. X.I. indicates the Xisha Islands, and N.I. indicates the Nansha Islands. The figure was made using MATLAB R2016b (http://www.mathworks.com).


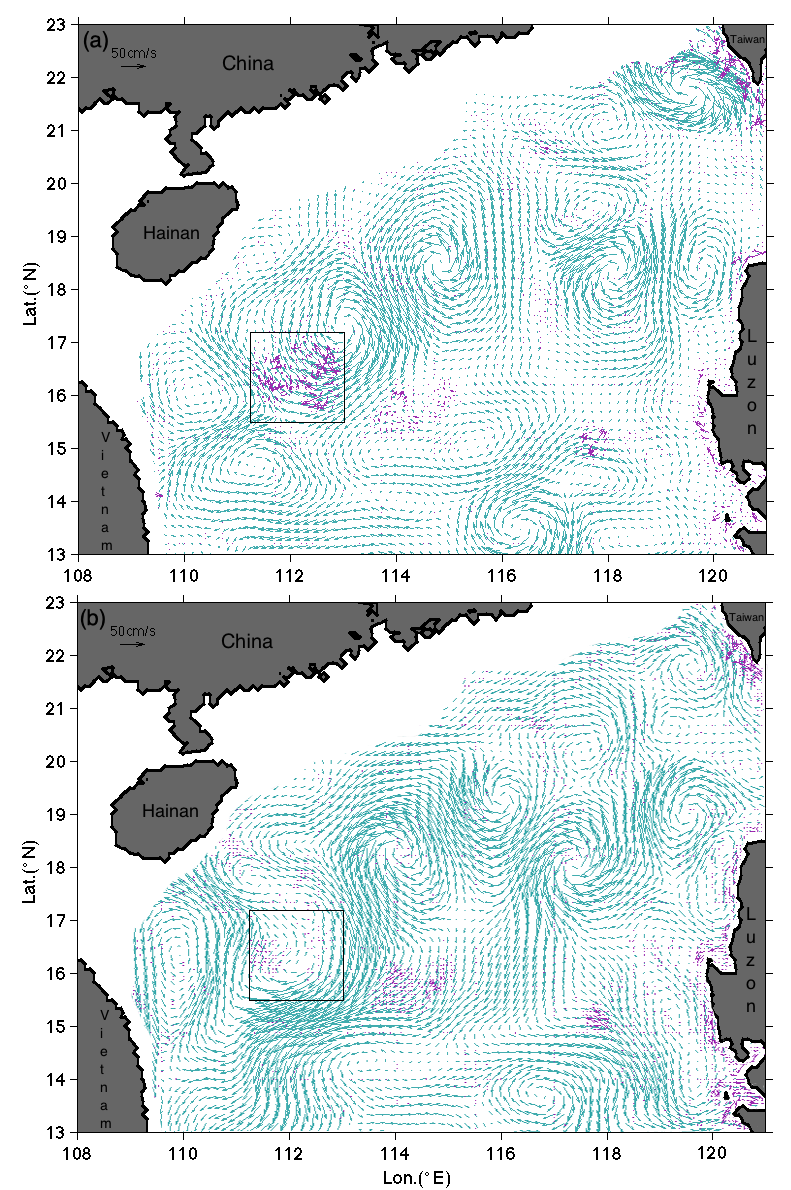


**Figure S3. Horizontal velocity field in the northern SCS.** The black box indicates the location of Xisha Islands, cyan arrows indicate mesoscale component, and purple arrows indicate the submesoscale components. Panela (a) shows that mesoscale eddy reach the Xisha Islands, accompanied by the energetic submesoscale currents on May 22, 2003. Panel (b) shows that a there is no mesoscale eddy near the Xisha Islands where the submesoscale currents are weak on June 01, 2003. The figure was made using MATLAB R2016b (http://www.mathworks.com).


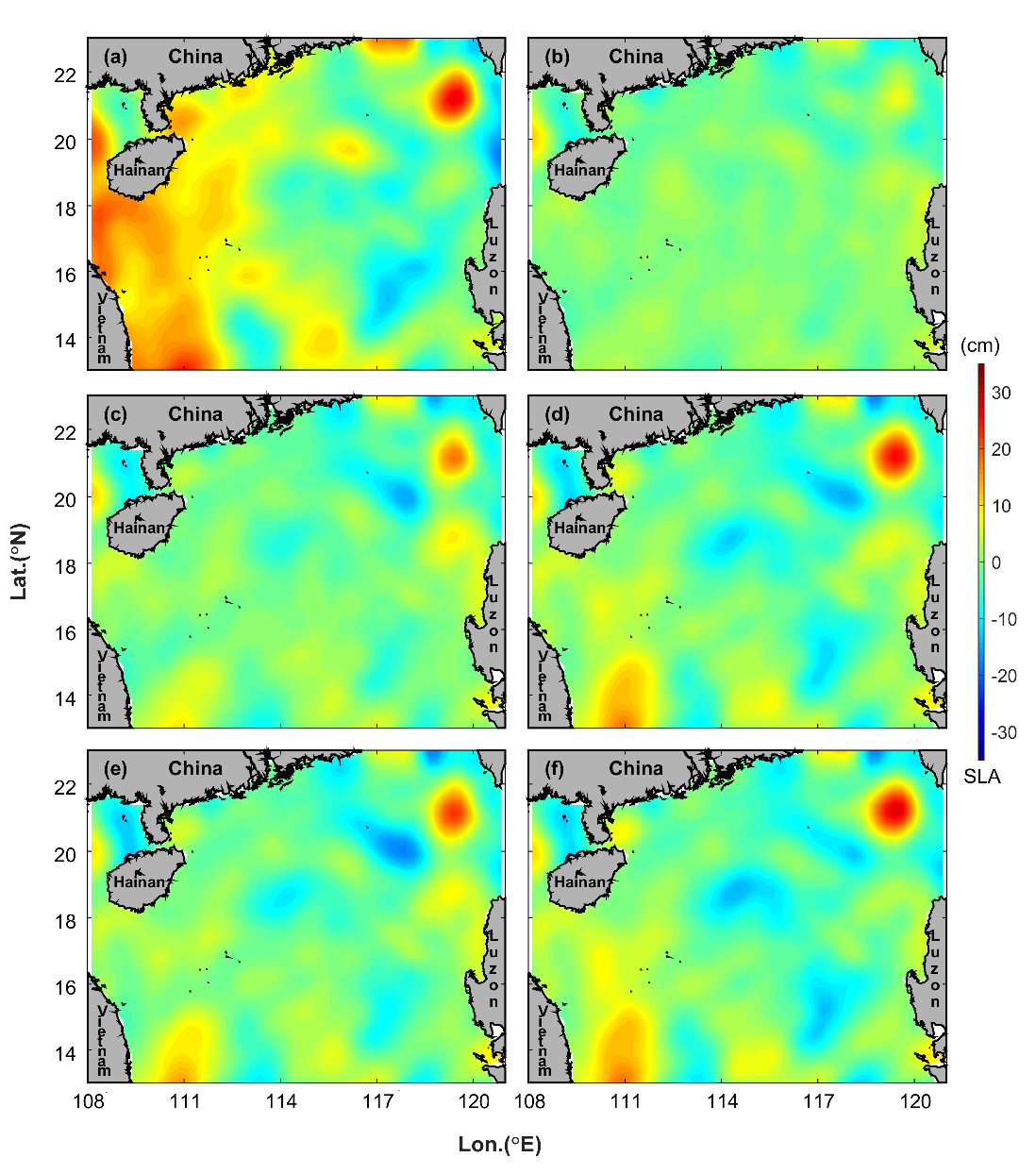


**Figure S4.** **SLA field on January 5, 2010.** Panel (a) shows the SLA field of the CMEMS product, and panels (b)-(f) show the computed SLA fields by removing 1-month to 5-month averages from the SSH field, respectively. The root mean square values between (a) and another panel, which is (b), (c), (d), (e), or (f), are 7.9, 7.3, 6.3, 6.9, and 7.0 cm, respectively. The figure was made using MATLAB R2016b (http://www.mathworks.com).


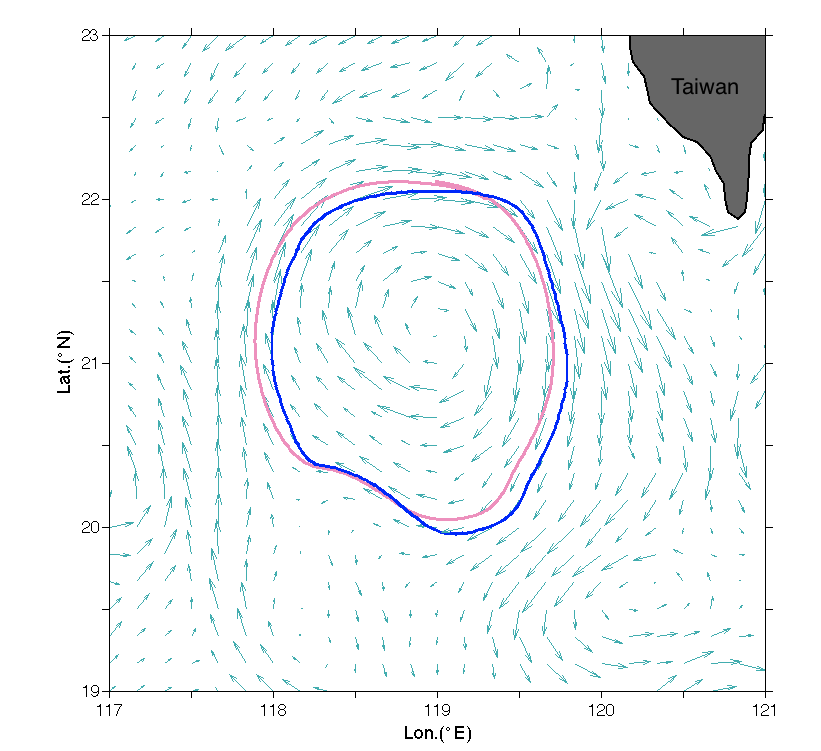


**Figure S5.** **Determination of eddy boundary in the surface layer.** Pink circle is determined from SLA field, and the blue one is from stream function. The cyan arrows indicate mesoscale currents. The figure was made using MATLAB R2016b (http://www.mathworks.com


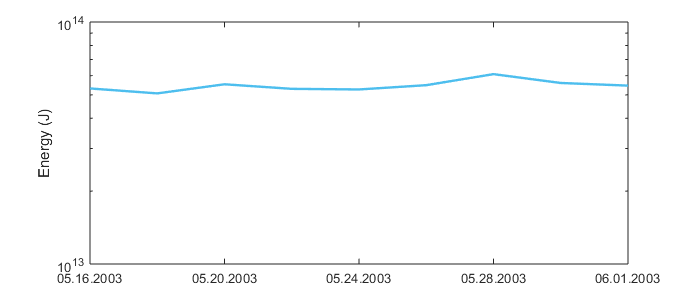


**Figure S6. Temporal variations of kinetic energy of mean flow from May 16 to June 1, 2003.**


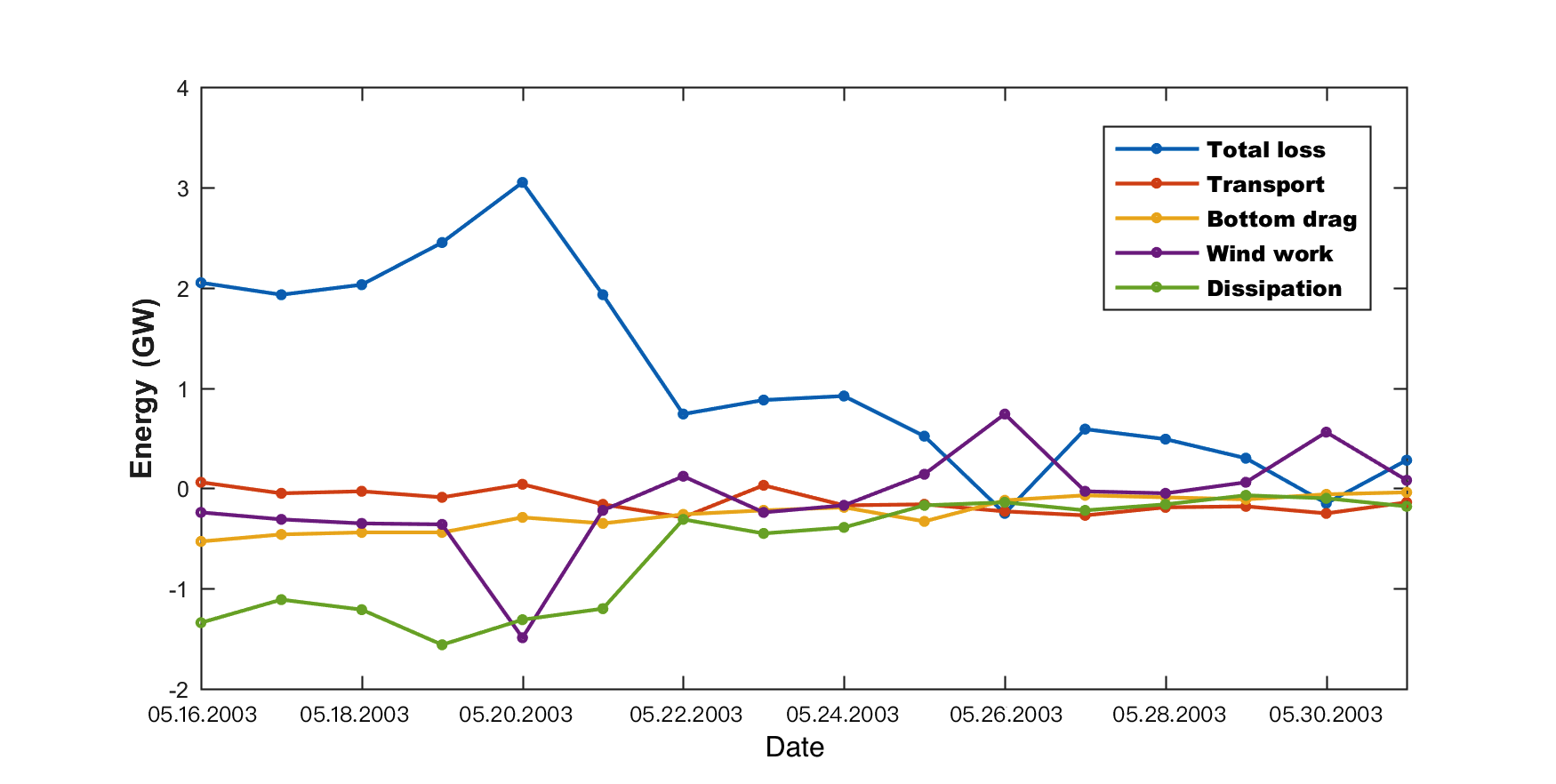


**Figure S7. Temporal variations of total EKE loss rate (**$\frac{\partial}{\partial t}\mathrm{EKE}$**) and the contribution terms.**

**
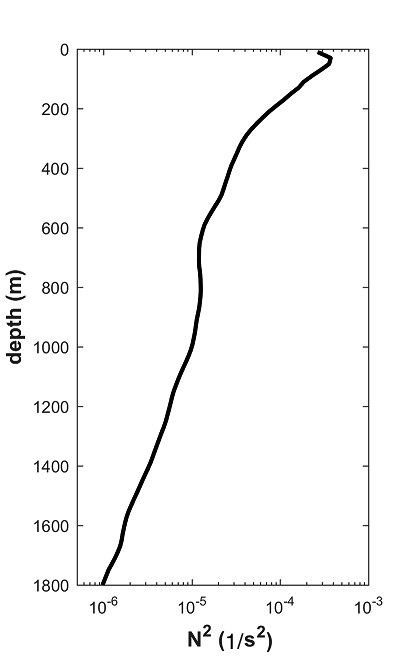
**

**Figure S8. Vertical profile of squared buoyancy frequency used to estimate eddy diffusivity.**

**Table S1.** The statistical results derived from 24-year altimeter observation (1993-2016), which indicate westward propagating eddies are dissipated around the Xisha Islands.

| No. | Start | | | End | | | Warm/Cold |
| --- | --- | --- | --- | --- | --- | --- | --- |
|  | Date/time  (dd.mm.yyyy) | Lon. (E) | Lat.  (N) | Date/time  (dd.mm.yyyy) | Lon. (E) | Lat.  (N) |  |
| 1 | 28.10.1995 | 117˚35’ | 19˚57’ | 21.01.1996 | 111˚47’ | 17˚27’ | Warm |
| 2 | 29.01.1996 | 116˚15’ | 19˚20’ | 30.03.1996 | 111˚17’ | 17˚25’ | Cold |
| 3 | 20.09.2000 | 116˚03’ | 19˚40’ | 12.11.2000 | 111˚30’ | 17˚04’ | Cold |
| 4 | 05.01.2010 | 119˚06’ | 21˚47’ | 06.05.2010 | 112˚35’ | 16˚50’ | Warm |
| 5 | 10.12.2011 | 119˚57’ | 21˚59’ | 23.04.2012 | 112˚15’ | 17˚39’ | Warm |
| 6 | 29.11.2013 | 118˚50’ | 21˚27’ | 10.03.2014 | 112˚25’ | 17˚15’ | Warm |
| 7 | 14.11.2015 | 118˚09’ | 21˚37’ | 30.01.2016 | 112˚05’ | 17˚32’ | Warm |
